# Supplementary material for: Cancer-associated DAXX mutations reveal a critical role for ATRX localization in ALT suppression
Source: bioRxiv. 2024 Nov 20:2024.11.18.624165. Preprint. [Version 1] doi: 10.1101/2024.11.18.624165 (PMC11722231; doi:10.1101/2024.11.18.624165)
Supplement: Supplement 1 [file media-1.pdf]

	F	R
L130R	CAATGAGCGCTGCACTGTTCTCAAGGCC	GTGCAGCGCTCATTGATGTAGACATAGAGC
L134P	CACTGTTCCCAAGGCCCACTCAGCCAA	GCCTTGGGAACAGTGCAGAGCTCATTGA
N153S	CACCTCCAGTGAGCCCTCTGGGAATAACCCTC	GGCTCACTGGAGGTGGTGGCGGCAG
S220P	ACCCAGACCCCGCATACCTGCAGGAGGC	ATGCGGGGTCTGGGTCATCCAATTCTGAGAG
Y222E	CTCCGCAGAACTGCAGGAGGCACGGTTG	TGCAGTTCTGCGGAGTCTGGGTCATCC
E225A	CCTGCAGGCGGCACGGTTGAAGCGTAAGC	CGTGCCGCCTGCAGGTATGCGGAGTCTGG
N268S	AGAGGTTAGCAGGCGCATTGAGCGGCTC	CGCCTGCTAACCTCTGGGTAGCGGGTGC
A297P	AGAAGGCACCTGCCCGACACAGCCTTG	GGGCAGGTGCCTTCTCTACAGCCCG
G321D	AGATGTGGACATCAGGTTACAGGAGCGACG	CTGATGTCCACATCTCGGAAGGCATCC
Q469H	CTGGAACACATGCAGGAGGGTCAGGAGG	CTGCATGTGTTCCAGATCCTCCTCCTC
D738V	GCTCTCAGTCTCTGATGACCCAGCTTTCTTGTAC	TCAGAGACTGAGAGCACGATGATCTCTTCTGG

Supplementary Table 1. DNA Oligonucleotides used to generate lentivectors for this study

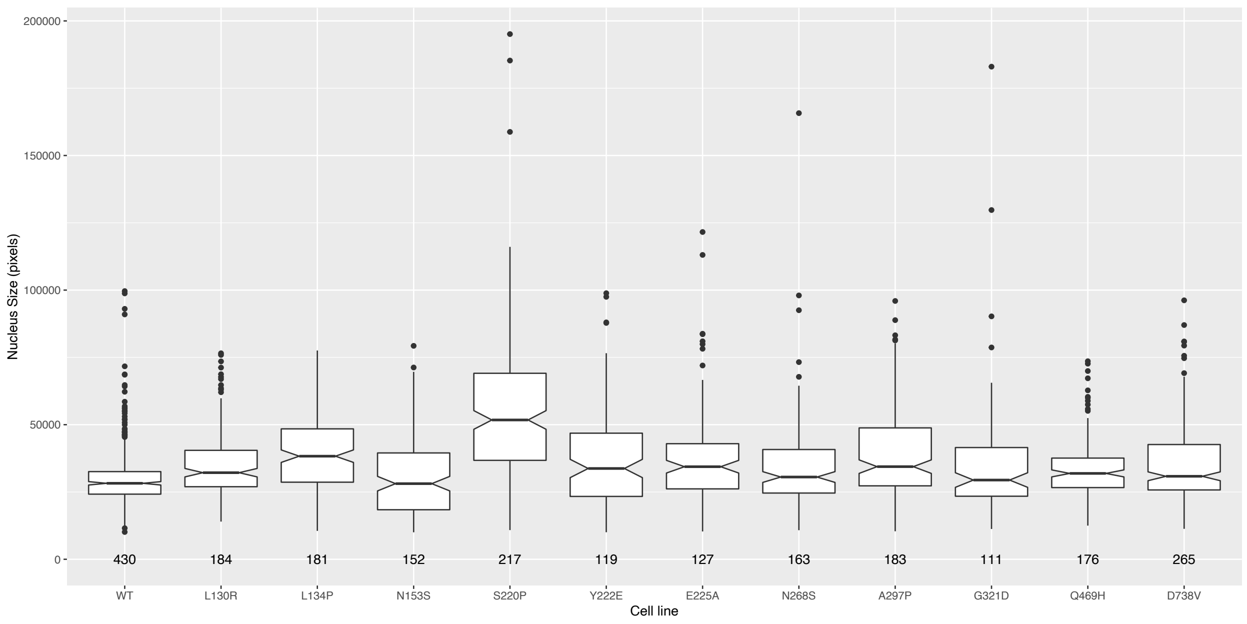

Supplementary Figure 1. Sizes of nuclei in DAXX variant stable lines. Larger nuclei in the S220P cell line suggest a genome duplication event.

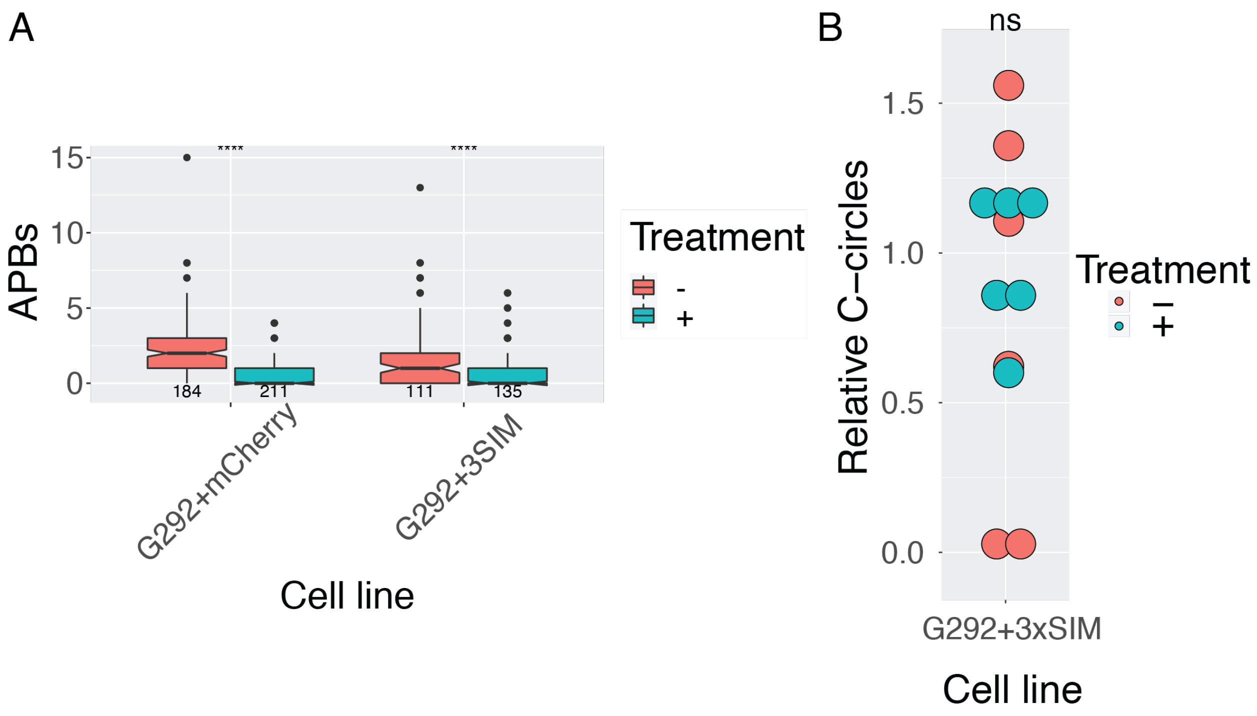

Supplementary Figure 2. APBs are sensitive to induction with doxycycline, but C-circles are not. A. Induction of expression of either mCherry or mCherry-3xSIM with doxycycline decreases APBs per cell significantly. B. Induction of mCherry-3xSIM does not change C-circle levels, implying the reduction in number of APBs does not reflect a *bona fide* change in ALT status.

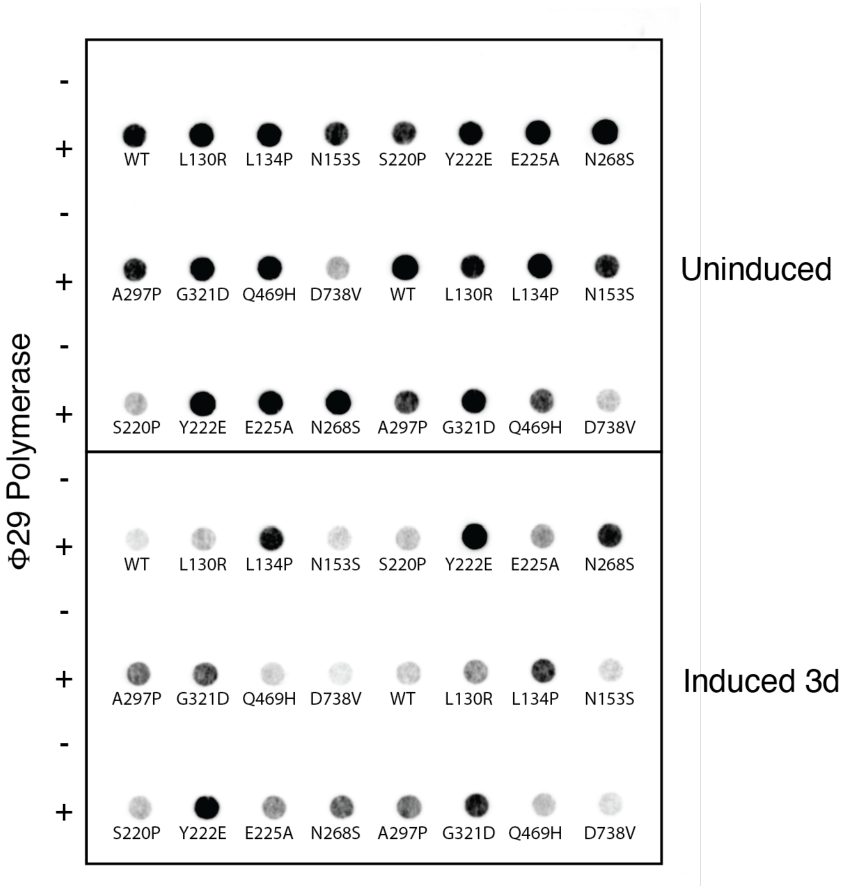

Supplementary Figure 3. Representative C-circle dot blot. Spot intensities were measured using ImageJ, and normalized to the median per-blot intensity for comparison across experiments.
